# Clinical Potentials of Methylator Phenotype in Stage 4 High-Risk Neuroblastoma: An Open Challenge

**DOI:** 10.1371/journal.pone.0063253

**Published:** 2013-05-22

**Authors:** Barbara Banelli, Domenico Franco Merlo, Giorgio Allemanni, Alessandra Forlani, Massimo Romani

**Affiliations:** 1 Tumor Genetics and Epigenetics, IRCCS AOU San Martino-IST, Genova, Italy; 2 Epidemiology, Biostatistics and Clinical Trials, IRCCS AOU San Martino-IST, Genova, Italy; Sapporo Medical University, Japan

## Abstract

Approximately 20% of stage 4 high-risk neuroblastoma patients are alive and disease-free 5 years after disease onset while the remaining experience rapid and fatal progression. Numerous findings underline the prognostic role of methylation of defined target genes in neuroblastoma without taking into account the clinical and biological heterogeneity of this disease. In this report we have investigated the methylation of the *PCDHB* cluster, the most informative member of the “Methylator Phenotype” in neuroblastoma, hypothesizing that if this epigenetic mark can predict overall and progression free survival in high-risk stage 4 neuroblastoma, it could be utilized to improve the risk stratification of the patients, alone or in conjunction with the previously identified methylation of the *SFN* gene (14.3.3sigma) that can accurately predict outcome in these patients. We have utilized univariate and multivariate models to compare the prognostic power of *PCDHB* methylation in terms of overall and progression free survival, quantitatively determined by pyrosequencing, with that of other markers utilized for the patients' stratification utilizing methylation thresholds calculated on neuroblastoma at stage 1–4 and only on stage 4, high-risk patients. Our results indicate that *PCDHB* accurately distinguishes between high- and intermediate/low risk stage 4 neuroblastoma in agreement with the established risk stratification criteria. However *PCDHB* cannot predict outcome in the subgroup of stage 4 patients at high-risk whereas methylation levels of *SFN* are suggestive of a “methylation gradient” associated with tumor aggressiveness as suggested by the finding of a higher threshold that defines a subset of patients with an extremely severe disease (OS <24 months). Because of the heterogeneity of neuroblastoma we believe that clinically relevant methylation markers should be selected and tested on homogeneous groups of patients rather than on patients at all stages.

## Introduction

Neuroblastoma (NB), a neoplasia derived from ganglionic precursors of the sympathetic nervous system, is the most common extra cranial solid tumor of infancy. This tumor is highly heterogeneous and its clinical behavior ranges from spontaneous regression to rapid and aggressive progression, metastatic spreading and poor outcome [1]. About half of the children with malignant NB have metastatic disease at diagnosis and, according to the International Neuroblastoma Staging System (INSS), are classified at stage 4 [2]. The guidelines set for by the International Neuroblastoma Risk Group (INRG) subdivide these patients into three categories (high, intermediate and low risk) depending on clinical and biological criteria [3]. This risk stratification is part of the clinical decisional tree and helps to choose the most suitable treatment.

In stage 4, high-risk NB approximately 80% of the patients experience rapid and fatal disease while the remaining subgroup performs well with most of the patients alive and free of disease 5 years after diagnosis [1]. This dramatic difference suggests that the prediction of outcome for these patients is still inaccurate. In perspective, the precise classification of NB patients into risk classes according to clinical and molecular parameters is of the utmost importance for the selection of the most effective treatment. In this respect, NB is one of the first tumors where a molecular marker, the amplification of the *MYCN* oncogene, has been utilized to choose the optimal therapeutic protocol [1], [4]. Nevertheless the identification of predictive biomarkers in NB is made difficult because of its clinical and biological heterogeneity.

The aberrant DNA methylation is considered a promising biomarker of outcome or response to treatment, and the potential clinical application of methylation analysis in cancer is actively investigated [5], [6], [7]. The aberrant and concordant methylation at multiple loci, known as CpG Island Methylator Phenotype (CIMP), was initially described in colorectal tumors [11], and is considered a potential predictive biomarker in cancer. In neuroblastoma, CIMP was originally associated to clinically distinct subgroups of patients by quantitative methylation analysis conducted on a series of patients at stages 1–4, assigned at different INRG risk groups and with different clinical and biological features [12], [13].

In univariate analyses CIMP had a strong predictive power on outcome and its prognostic power was entirely recapitulated by the methylation of 17 genes of the Protocadherin B cluster (*PCDHB*) that is the most informative member of the Methylator Phenotype in this tumor [14]. However, when other biomarkers commonly used in the clinical practice were included in a multivariate model, CIMP lost its prognostic power likely because the overall number of patients examined was insufficient or because of the known heterogeneity of NB [14].

In a subsequent independent study, in a multivariate analysis that included age at diagnosis, stage and *MYCN* amplification, CIMP was found to have a strong influence on disease-free survival, but not on overall survival [15], [16].

In view of a possible translational application of methylation of *PCDHB* cluster to improve risk stratification in stage 4 NB patients at high risk, we have evaluated the predictive power of *PCDHB* methylation by quantitative analysis in stage 4 NB at high risk, the most common mode of presentation of this disease which represents a clinically relevant problem in terms of accurate patients stratification and improvement of outcome.

For comparison within the metastatic stage 4, we have included a group of stage 4 patients at intermediate/low risk of progression. In retrospective studies, we identified the *SFN* gene (14.3.3sigma) as a methylation target in aggressive NB [8], [9] and found that quantitative methylation differences in *SFN* discriminated high-risk stage 4 patients with poor survival from those, at the same stage and in the same risk group, with favorable outcome independently from *MYCN* amplification, treatment, clinical response, histology and age at diagnosis [10].

We have conducted the present study in the same series of patients previously utilized for the *SFN* analysis considering all biological and clinical features currently used for patients' stratification including *MYCN* amplification.

The rationale of our work was to determine if *PCDHB,* alone or in conjunction with *SFN* could improve the risk stratification in these patients as a preliminary step in order to select members of a classifier predictive of outcome in stage 4, high-risk NB.

## Methods

### Ethics statement

The Ethics Committee of the Giannina Gaslini Children Hospital of Genoa approved the collection, the storage in the Neuroblastoma Tissue Bank and the utilization of this material. Written informed consent was obtained for all patients from their parents or legal representative.

### Planning of the study

In the present study we have analyzed a total of 121 NB patients at stage 4 diagnosed between 1990 and 2004. The clinical endpoints examined were the overall survival (OS) at 60 months and the progression free survival (PFS) in relation with the level of methylation of the genes examined.

To stratify the patients into risk groups we have utilized the criteria of the International Neuroblastoma Risk Group (INRG) Classification System [3]. Accordingly, 106 stage 4 patients were considered at “high risk”: 100 patients were older than 18 months, and 6 were younger than 18 months at diagnosis but their tumor presented *MYCN* amplification. Within the stage 4 high-risk group we considered the patients as “short survivors” (HR-SS) if they died for disease within 60 months (N = 83) from diagnosis and as “long survivors” (HR-LS) if they survived more than 60 months (N = 23). We conducted the methylation analysis on high-risk patients first on a training set of 41 patients and then on a validation set of 65 patients.

As control group we have included 15 stage 4 patients at intermediate and low risk (I/LR: below 18 months of age at diagnosis, *MYCN*-single copy), all alive and free of disease 60 months after diagnosis.

The clinical characteristics of the study groups are reported in Table 1. The patients examined for the present study are the same described in a previous report [10] with the exception of 17 patients whose tumor samples were no longer available.

**Table 1:** Summary of the clinical characteristics of the patients included in the study.

		Training Set		Validation Set		Control
		Short Surrvivors	Long Survivors	Short Surrvivors	Long Survivors	Long Survivors
		High Risk		High Risk		Intermediate and Low Risk
Patients N		31	10	52	13	15
Age at diagnosis: Months mean (SD)		44.5 (20.9)	58.6 (49.9)	56.7 (SD)	51.9 (SD)	7.86 (2.92)
Histology N	NB	25	6	49	11	12
	GNB	2	3	1	1	2
	NS	4	1	2	1	1
*MYCN* amplification N (%)		14 (43.7)	0 (0)	18 (35.3)	5 (38.5)	0 (0)
Ferritin levels N (%)	<92ng/ml	5 (16.1)	7 (60)	5 (9.6)	3 (23.2)	15 (100)
	≥92ng/ml	24 (77.4)	3 (40)	37 (71.1)	9 (69.2)	0 (0)
	ND	2 (6.5)	0 (0)	10 (19.2)	1 (7.7)	
PFS median (months)		16	73.5	21	72	81.53
OS median (months)		25.13	84.1	30.5	89	87.67

NB: Neuroblastoma.

GNB: Ganglioneuroblastoma.

NS: not specified neuroblastic tumor.

ND: not done.

### Methylation analysis

We retrieved the tumor DNA from the Italian Neuroblastoma Tissue bank [10]. DNA (1 μg) was modified by sodium bisulfite treatment and the level of methylation for *SFN* and *PCDHB* was determined by pyrosequencing, a sequence-by-synthesis technique that allows the quantitative determination of the level of methylation of each CpG doublets within a target sequence [17]. The primers were designed with the Pyrosequencing Assay Design Software (Qiagen, Milano, Italy).

The pyrosequencing methylation assays for *PCDHB* cluster and *SFN* have been previously described in details [10], [18]. We have conducted the sequencing analysis utilizing the Pyro Q-CpG software (version 1.0.9) and the results of the pyrosequencing passed the quality controls built in the instrument. Blank reactions (for PCR and pyrosequencing) have been included in each assay to exclude cross contaminations. The specificity of the primers was determined in PCR reactions conducted on unmodified DNA to ensure that only the modified DNA was amplified.

### Statistical analysis

The mean methylation values of the CpG doublets included in the target sequences were measured for *PCDHB* cluster and these values were considered for the statistical analysis.

The correlation of the percentage of methylation between *SFN* and the *PCDHB* cluster was assessed by computing the Pearson's linear correlation coefficient.

The statistical differences in the methylation levels were determined by the Student t-Test.

We defined overall survival as the time elapsed from the date of diagnosis and the date of death. Only cancer-related deaths were considered. Patients that survived were censored at the last date they were reported to be alive. Progression free survival was calculated from the date of diagnosis to the date of relapse, as reported in the clinical records.

For survival analysis, besides the already defined levels of methylation thresholds for *SFN* (85%) [10] and for the *PCDHB* cluster (40%) [18], we determined, according to the Receiver Operating Characteristic (ROC) curves, a new methylation threshold for *PCDHB* (58.3%) specific for high risk stage 4 patients that could distinguish between long and short survivors. Survival curves were computed according to the Kaplan-Meier method and were compared using the log-rank test.

The Cox proportional-hazards multiple regression model was used to study the relation between DNA methylation of *SFN* and *PCDHB* and the overall and progression free survival. We included in the regression model the following factors known as clinically relevant in the INGR classification of stage 4 high risk patients: age at diagnosis (<18 months or ≥18 months), *MYCN* amplification (single copy or amplified) and ferritin serum level (<92 or ≥92 ng/ml).

A p value below 0.05 was considered statistically significant. Statistical analyses were done with the IBM® SPSS® Statistics 20 software.

## Results

We have analyzed by quantitative pyrosequencing the methylation of the *PCDHB* cluster in 121 NB samples derived from patients at stage 4 divided into training and validation sets of high-risk patients and in a control set of intermediate/low risk patients. We have matched these data with a previous *SFN* methylation analysis conducted on the same patients [10]. The clinical characteristics of the patients are summarized in Table 1 and the complete clinical and biological data are shown in Table S1.

Similarly to what we have observed for the *SFN* gene [10], the overall distribution of the *PCDHB* methylation levels observed in our series of high risk stage 4 patients did not follow the bimodal distribution described by Abe et al [14] for a population of patients with neuroblastoma representative of all stages of the disease ( Figure S1).

We have evaluated the correlation between methylation levels of the *SFN* gene and of the *PCDHB* cluster by computing the Pearson's linear correlation coefficient in high risk (HR) and intermediate/low risk (I/LR) patients and subdividing the high risk patients in high risk-long and -short survivors groups (HR-LS and HR-SS, respectively) as defined in methods section. The result of this analysis are presented in Figure S2 and show that methylation levels of *PCDHB* and *SFN* were poorly correlated.

**Figure 1:**
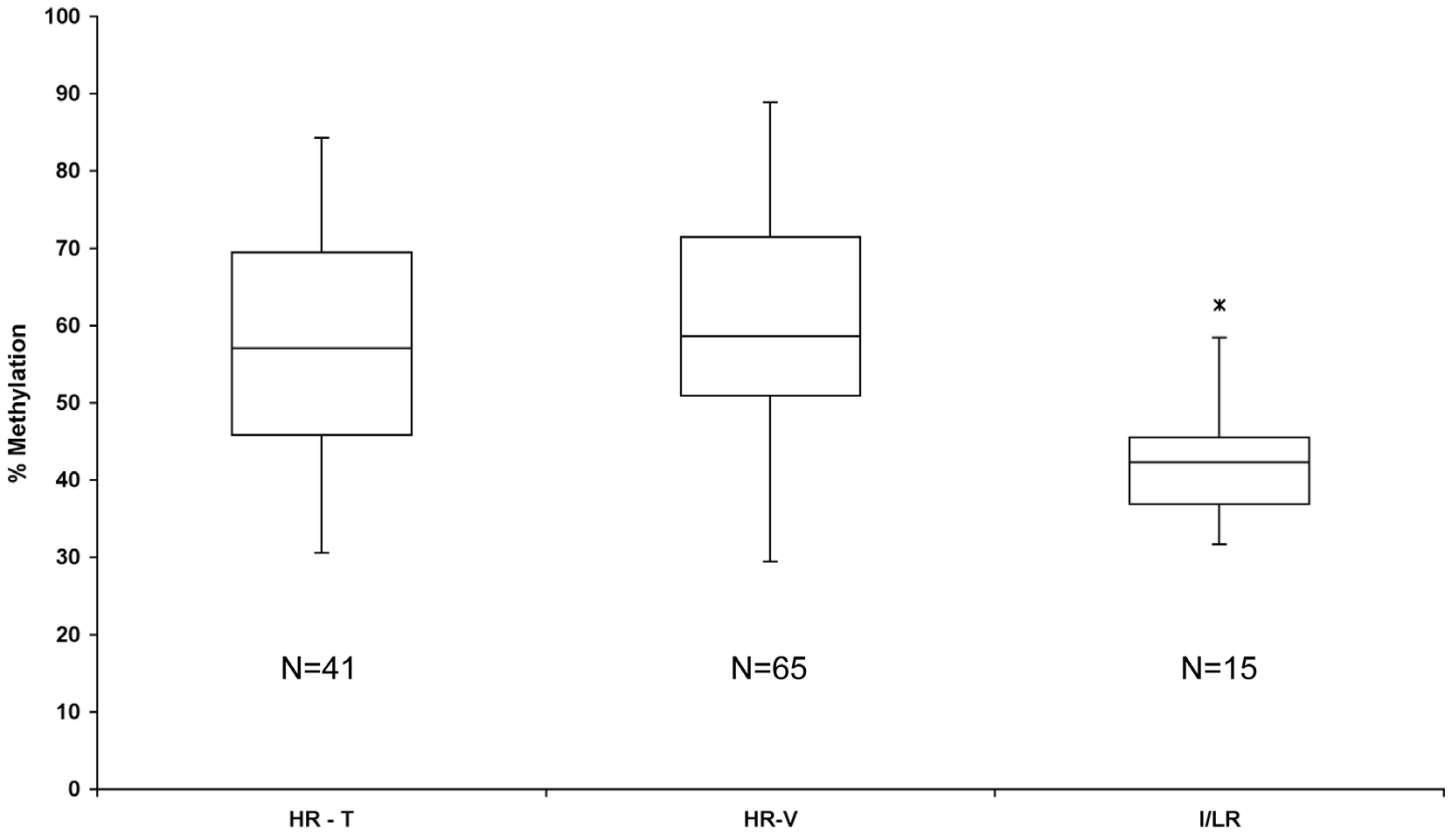
Distribution of methylation values for *PCDHB* cluster in high-risk patients subdivided in training (HR-T) and validation (HR-V) and compared with intermediate and low-risk patients (I/LR). The lower and upper boundaries of each box are the 25th and 75th percentile. Black bars represent median values; wiskers are the smallest and largest values that are not outliers (defined as larger than 1.5 and smaller than 3 box lengths from 25th and 75th percentiles). The black star is the upper outlier.

The comparison between the high risk (training and validation sets) and intermediate/low risk stage 4 patients showed that the mean methylation levels of the *PCDHB* cluster were significantly different between the two risk groups (Figure 1; *PCDHB* mean values: HR-T = 56.8, HR-V = 60.4 I/LR  = 41.93, p<0.001). This result indicated that the methylation of the *PCDHB* cluster, as also observed also for *SFN* gene [10], was associated with risk stratification in stage 4 patients.

**Figure 2:**
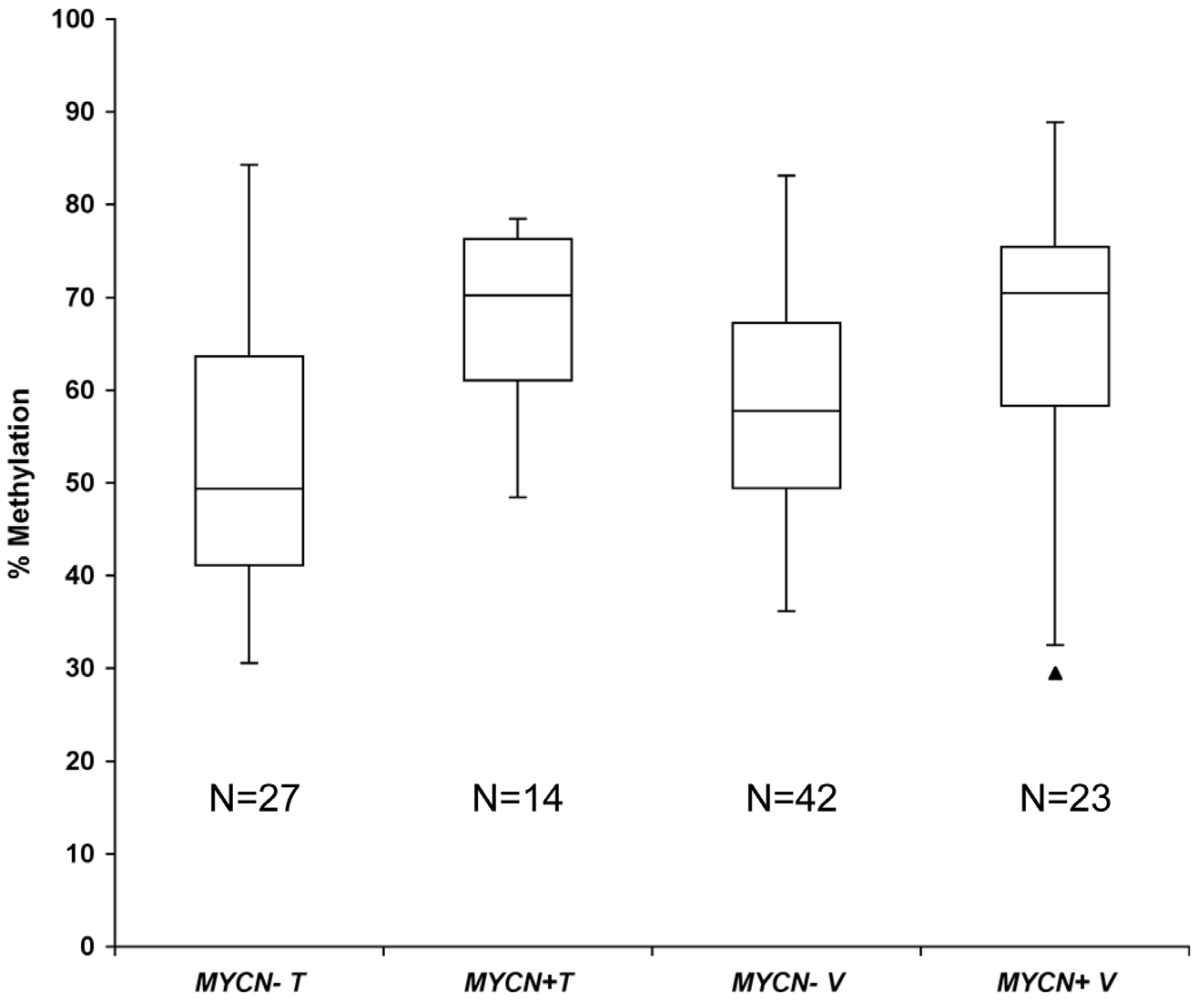
Distribution of methylation values for *PCDHB* in HR patients subdivided according to the *MYCN* amplification status (+: amplified; −: single copy) in the training (T) and in the validation set (V).

The relation between DNA methylation and *MYCN* amplification in NB is controversial and it is still an open question [8], [10], [19], [20]. According to our previously published data, hypermethylation of *SFN* in stage 4 high risk NB patients was independent from *MYCN* amplification [10]. In a large series of NB patients at stages 1–4, methylation of the *PCDHB* cluster was found to be related to *MYCN* amplification; this association remained true also in our series of stage 4 high-risk patients (Figure 2, p<0.001 and p = 0.019 in training and validation sets, respectively).

It has been previously shown that the methylation threshold of 40% for the *PCDHB* cluster, determined by MSqPCR assay, predicts overall survival and progression free survival in NB patients at stages 1–4 [14], [16]. In a previous report we described a methylation pyrosequencing assay for *PCDHB* cluster and showed that it provided similar results to MSqPCR when tested on a set of patients selected to be at the extreme ends of the INRG classification system in terms of outcome and disease progression [18]. Interestingly, the threshold determined by pyrosequencing was essentially identical to that found by MSqPCR on a distinct patient series [14] (39.15% versus 40% respectively).

The Kaplan-Meier plot for high-risk stage 4 patients categorized into two groups according to the 40% threshold of methylation for *PCDHB*, showed no significant association with progression free survival and overall survival (Figure S3).

We have previously shown that in NB, higher methylation levels were associated with higher aggressiveness [8]. In agreement with this finding, we observed that in high-risk, stage 4 patients that survived less than 24 months, the methylation level of SFN was significantly higher than that of the patients that died between 25 and 60 months (Figure S4).

**Figure 3:**
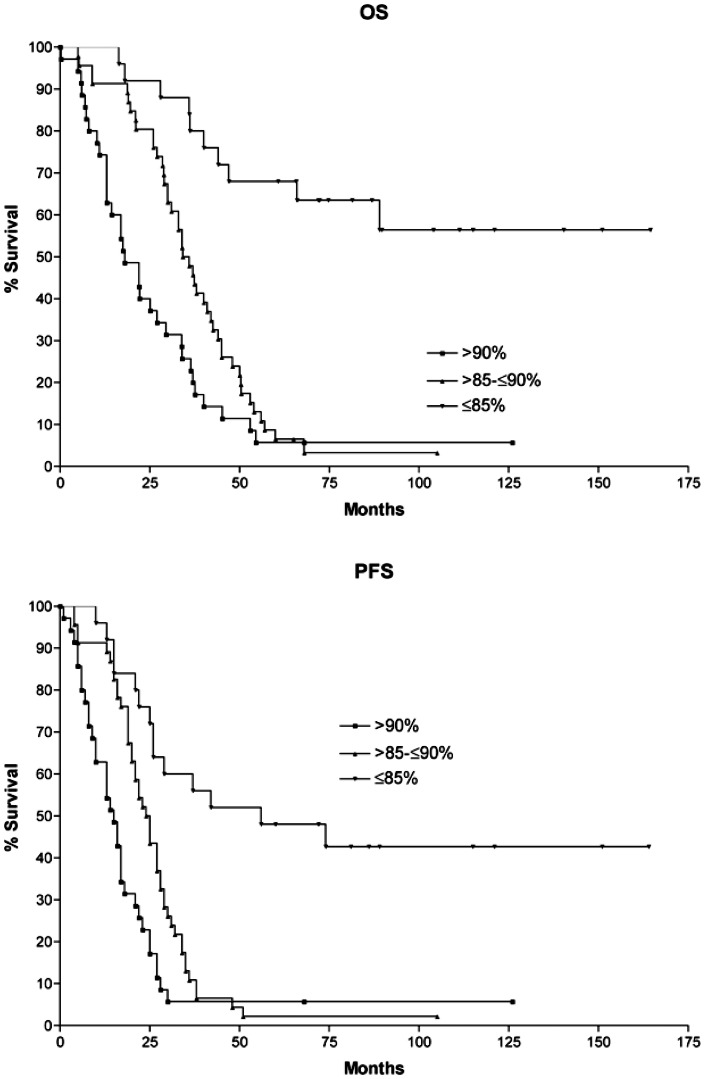
Kaplan-Meier estimates of OS and PFS of the 106 High-Risk patients assigned to groups according to the methylation level of the *SFN* gene: ≤85% (N = 27); >85%–≤90% (N = 44) and >90% (N = 35). Hazard Ratio and *p* values are reported in Table S2.

Accordingly, a second threshold of methylation for the *SFN* gene (90%) identified the patients with a median survival halved from 36 to 18 months compared to patients with methylation comprised between 85 and 90% (Figure 3 and Table S2). Thus, methylation levels above 90% appeared to characterize a subset of patients with extremely aggressive disease and were suggestive of a linear trend of *SFN* methylation associated with poor outcome in NB at high risk (Log-rank test for trend P<0.0001 for OS and PFS; the same results were obtained also analyzing the training and validation set separately even if the size of the two groups was relatively small, data not shown).

**Figure 4:**
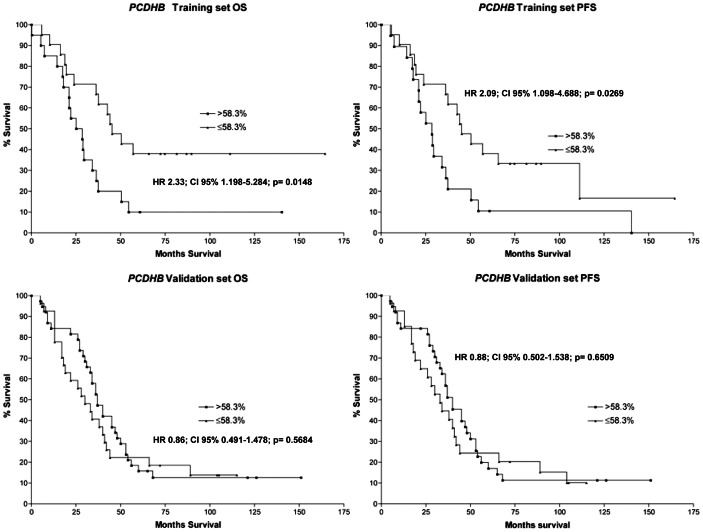
Kaplan-Meier estimates of OS and PFS of the High-Risk patients assigned to groups according to the 58.3% thresholds of methylation for *PCDHB* (Training set: ≤58.3% N = 21; >58.3% N = 20; Validation set: ≤58.3% N = 27; >58.3% N = 38). The Hazard Ratio (HR) and the corresponding *p* values (Cox Long-Rank test) are reported.

To determine if methylation of *PCDHB* followed a similar trend we calculated by ROC analysis a second threshold of methylation for the *PCDHB* cluster predictive of outcome taking into consideration only the high-risk patients at stage 4. The resulting thresholds (58.3%) were the best compromise between specificity (80%) and sensitivity (58%). According to this new threshold, methylation of the *PCDHB* cluster in the training set showed a significant association with progression free and overall survival (p = 0.0269 HR = 2.09 and p = 0.0148 HR = 2.33 respectively). However, this association was not maintained in the validation set (OS: p = 0.5684 HR = 0.86; PFS: p = 0.6509 HR = 0.88) (Figure 4).

Finally, the possible prognostic power of *PCDHB* cluster methylation was compared to that of the *SFN* gene in a multivariate model to take into account the clinically relevant parameters considered for the risk stratification of stage 4 NB patients [3]. In this stepwise analysis the methylation of *PCDHB* cluster was excluded by the model before the *MYCN* amplification and ferritin serum level while *SFN* remained a statistically significant parameters to predict survival (Table 2).

**Table 2:** Cox regression analysis in High Risk stage 4 NB patients subdivided according to the methylation thresholds of *SFN* gene and *PCDHB* cluster (validation set).

		Overall Survival			Progression Free Survival		
		p	HR	95.0% CI	p	HR	95.0% CI HR
Full Model	*PCDHB* methylation ≤58.3% >58.3%	.772	.910	.479–1.727	.695	.883	.473–1.647
	*SFN* methylation ≤85% >85%	.005	3.030	1.399–6.566	.017	2.455	1.173–5.137
	Age at diagnosis <18 months ≥18 months	.799	1.186	.319–4.411	.017	2.455	1.173–5.137
	Ferritin serum levels <92ng/ml ≥92ng/ml	.166	1.983	.753–5.222	.233	1.726	.704–4.232
	*MYCN* Single copy Amplified	.680	.863	.430–1.733	.634	.844	.419–1.698
Final Model	*SFN* methylation ≤85% >85%	.005	2.947	1.388–6.258	.021	2.330	1.137–4.774

## Discussion

Specific methylation signatures predictive of outcome were identified in NB and contributed to the definition of the CIMP in this tumor [14], [15].

The initial characterization of the methylator phenotype in NB was carried out in a series of patients representative of the entire spectrum of this disease and thus at all INSS stages and assigned at different risk groups [14]. The heterogeneity in this patients' series made difficult to reach a sufficiently large number of cases to be analyzed in a robust multivariate model that included the biomarkers utilized in the clinical practice. From this pioneering study, the methylation of the *PCDHB* cluster above the established threshold of 40% was correlated with the reduced patients' survival and was considered the most informative member of CIMP in NB [14], [15], [16]. Interestingly, lower levels of *PCDHB* methylation are associated with a less aggressive behavior not only in NB but also in Wilm's and breast cancer [21], [22] indicating that this biomarker could be relevant in many tumor types.

In a subsequent neuroblastoma study, CIMP was proven to be a predictor of progression-free survival superior to *MYCN* amplification, stage and age at diagnosis [16]. In a independent report, utilizing a different technical approach, a similar level of *PCDHB* methylation distinguished patients at the opposite ends of the INRG classification system: the low-stage patients at favorable outcome and the stage 4 patients at unfavorable outcome [18].

Because of the known clinical and biological heterogeneity of neuroblastic tumors, we hypothesized that if stage-specific NB biomarkers are used, it could be possible to stratify more precisely the patients and identify subgroups of patients with distinct clinical characteristics. Indeed, we believe that the analysis of a homogeneous set of patients could give a clear answer about the potentiality of the methylation of *PCDHB* cluster to predict the outcome in the most aggressive group of neuroblastoma: the high risk at stage 4.

We demonstrated that the methylation threshold of 85% for *SFN* is a strong and independent predictor of outcome in high-risk, stage 4 patients [10]. Along this line we have now determined the methylation level of *PCDHB* in the same clinical series in the attempt to define if the prognostic power of *PCDHB* and *SFN* methylation could be increased by combining the information derived from the two biomarkers and if they were detecting the same or distinct subgroups of patients.

The weak correlation between *PCDHB* and *SFN* methylation observed in our study immediately suggested that the two biomarkers identify partially overlapping but not identical patients subgroups. In stage 4, as already observed for *SFN*, the *PCDHB* cluster showed higher methylation in high-risk respect to the intermediate and low risk subgroups indicating that the level of methylation of these genes followed the risk stratification set by the INRG. Nevertheless, the methylation threshold of *PCDHB* (40%) previously defined as predictive of outcome in NB at stages 1–4 did not predict outcome when only stage 4 high-risk patients were considered.

CIMP in cancer is generally associated with a worse prognosis (reviewed in [13]) although examples of improved outcome were described [23], [24]. In neuroblastoma, a higher number of methylated genes [25] or higher methylation levels in specific gene signatures [8], [14] are predictive of poor prognosis.

In a study conducted on stage 4 high-risk patients, we demonstrated the strong association between the level of methylation of *SFN* and outcome [10]. This association is now further strengthened by the observation that patients with methylation levels over 90% had a median survival time halved respect to patients with the methylation level between 85 and 90% (18 vs. 36 months).

In view of this result we hypothesized that the *PCDHB* methylation threshold predictive of outcome in high risk, stage 4 patients could be different from that calculated on the entire NB patients' population. Therefore, by ROC analysis we determined a second, higher, threshold of methylation only from stage 4 patients at high risk and showed that, in the training set, it could significantly distinguish two groups of patients according to their OS and PFS. However this threshold lost its predictive power when assayed on the validation set of patients.

Furthermore, a multivariate model that included the most relevant factors that predict outcome in this group of patients, underlined the predictive role of *SFN*. Overall these results indicate that, even if *PCDHB* methylation can correctly subdivide stage 4 patients in distinct risk groups, it is not a prognostic indicator in stage 4 high-risk neuroblastoma patients.

The conflicting results on the prognostic power of *PCDHB* cluster and of the *SFN* gene methylation in stage 4 high-risk patients probably reflect the different methodological approaches to examine these markers. *SFN* was selected through a candidate gene approach and validated in a retrospective study specifically focused on high risk stage 4 patients [8], [10], while the *PCDHB* cluster was identified by MS-RDA, a subtractive genome-wide analysis conducted between *MYCN* amplified NB cell lines and primary tumors with good prognosis [14]. It is thus possible that this latter approach has favored the selection of genes methylated in *MYCN* amplified tumors. This could explain the strong relation between CIMP and *MYCN* amplification observed in this and in previous studies [16]. In this respect the methylation of the *CYP26C1* gene, another member of CIMP selected in the same study [14], although highly predictive of outcome in univariate analysis, was highly correlated to *MYCN* amplification in stage 4 high risk patients and was selected out in a multivariate model that includes *MYCN* amplification as confounder [9], [10]. Similarly, *PCDHB* methylation in a stepwise Cox-regression model was the first marker to be excluded before *MYCN* amplification. Overall these results indicate that, *MYCN* amplification and CIMP, as defined in previous studies [14], [16], are linked phenomena and that the predictive power of CIMP observed in patients at stages 1–4, might be partially absorbed by *MYCN* amplification in high-risk stage 4.

Similarly to *SFN* [10], the distribution of *PCDHB* methylation values in high-risk stage 4 patients, was shifted toward higher levels of methylation. This result suggests that high-risk patients at stage 4 could be enclosed in the right end of the bimodal distribution described in a neuroblastoma population where all stages were represented [14]. According to this hypothesis it is understandable why biomarkers selected from the neuroblastoma general populations could not reliably separate patients with divergent outcome in a high-risk subgroup [26].

Approximately 50% of the NB patients have metastatic disease at diagnosis and hence are classified at stage 4; the majority of them are at high risk of progression although the prediction of their outcome is still imprecise. Thus, patients in this group are those likely to benefit more from a marker predictive of prognosis to improve their risk stratification. According to our data, *SFN* methylation, differently from *PCDHB*, can identify patients at lower or much higher risk within this group of patients opening the possibility to design tailored therapies.

Many different methylation markers were found to predict with variable accuracy the outcome of NB patients. We believe that the transfer of these markers “from the bench to the bedside”, considering the great heterogeneity in neuroblastoma, could be more easily obtained focusing on more homogeneous groups of patients and carefully taking into account, in a multivariate approach, all the relevant clinical and biological characteristics of that particular set of patients.

## Supporting Information

Figure S1 **Distribution of** ***PCDHB*** **cluster methylation in tumor samples from High Risk stage 4 patients (Long Survivors in red and Short Survivors in blue).** Histograms represent the number of cases according to the percentage of *PCDHB* cluster methylation.
(TIF)

Figure S2 **Correlation analysis between the mean methylation values of** ***PCDHB*** **and** ***SFN*** **in stage 4 NB patients subdivided according to risk class and survival.**
(TIF)

Figure S3 **Kaplan-Meier estimates of OS and PFS of the High-Risk patients assigned to groups according to the 40% thresholds of methylation for** ***PCDHB*** **(Training set: ≤40% N = 7; >40 N = 34).** The Hazard Ratio (HR) and the corresponding *p* values (Cox Long-Rank test) are reported.
(TIF)

Figure S4 **Distribution of methylation values for** ***SFN*** **in HR-SS patients subdivided in patients that died within 24** **months and between 25 and 60** **months from diagnosis.** Black stars and black triangles are the upper and lower outliers, respectively.
(TIF)

Table S1 **Clinical characteristics of the patients and methylation analysis.** For each patient are indicated the most relevant parameters as reported in the clinical records and the mean methylation values of the *PCDHB* cluster and of the *SFN* gene.
(XLS)

Table S2 **OS and PFS in High Risk stage 4 NB patients according to** ***SFN*** **methylation levels.**
(DOC)
