# Supplementary figures and images for: Clinical Potentials of Methylator Phenotype in Stage 4 High-Risk Neuroblastoma: An Open Challenge
Source: PLoS One. 2013 May 22;8(5):e63253. doi: 10.1371/journal.pone.0063253 (PMC3661569; doi:10.1371/journal.pone.0063253)

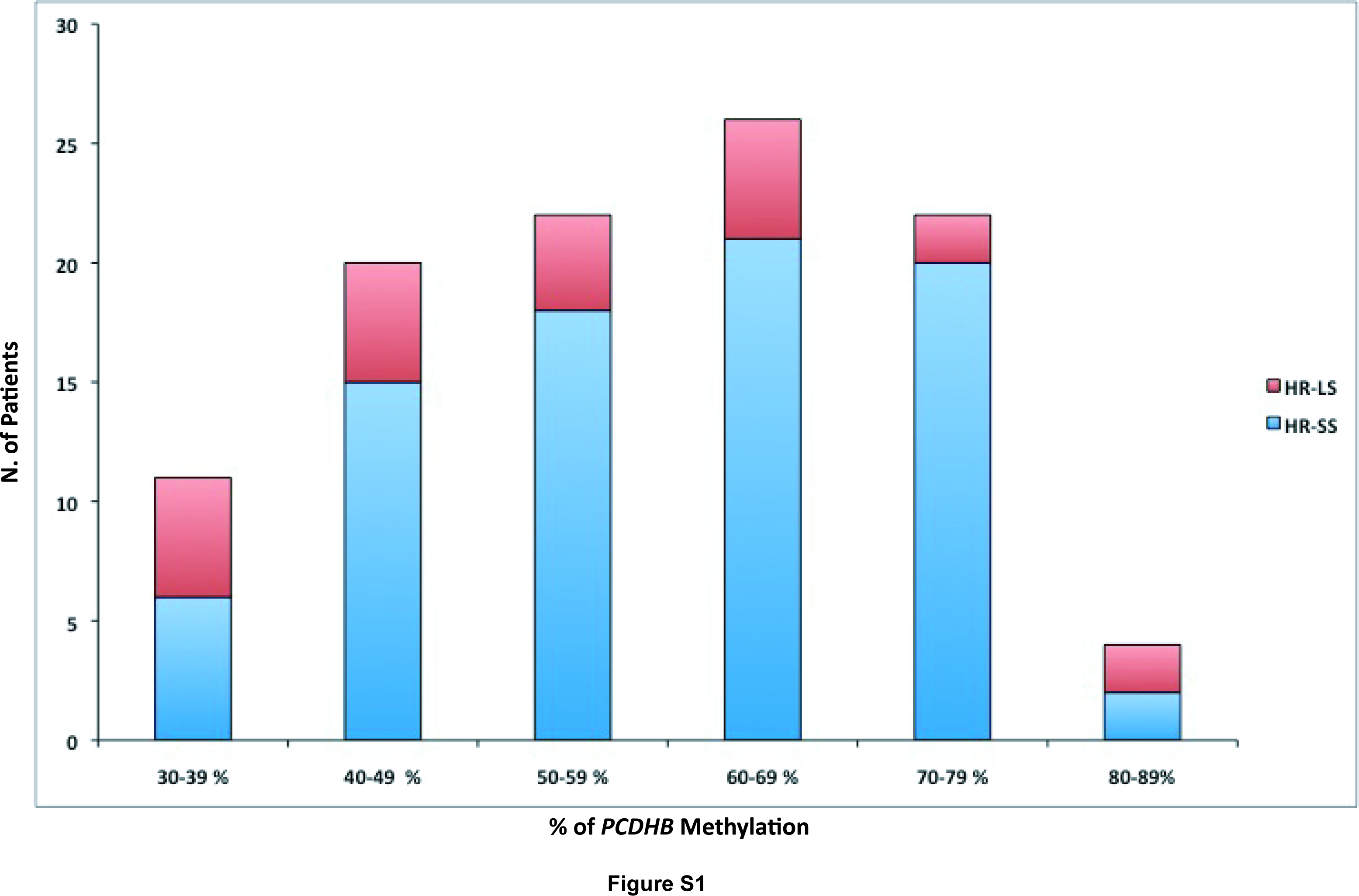

Supplement: Figure S1 — Distribution of PCDHB cluster methylation in tumor samples from High Risk stage 4 patients (Long Survivors in red and Short Survivors in blue). Histograms represent the number of cases according to the percentage of PCDHB cluster methylation. (TIF) [file pone.0063253.s001.tif]

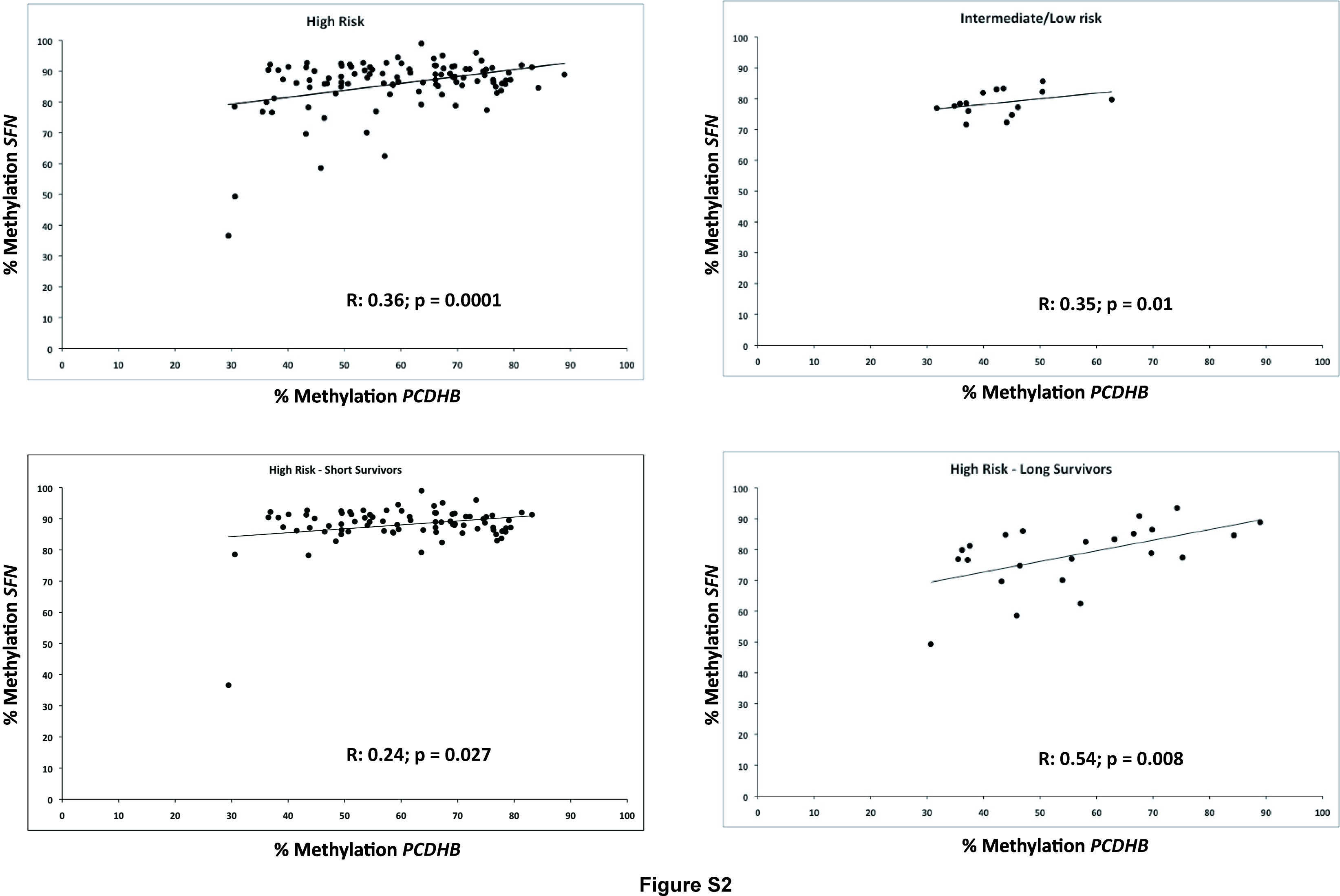

Supplement: Figure S2 — Correlation analysis between the mean methylation values of PCDHB and SFN in stage 4 NB patients subdivided according to risk class and survival. (TIF) [file pone.0063253.s002.tif]

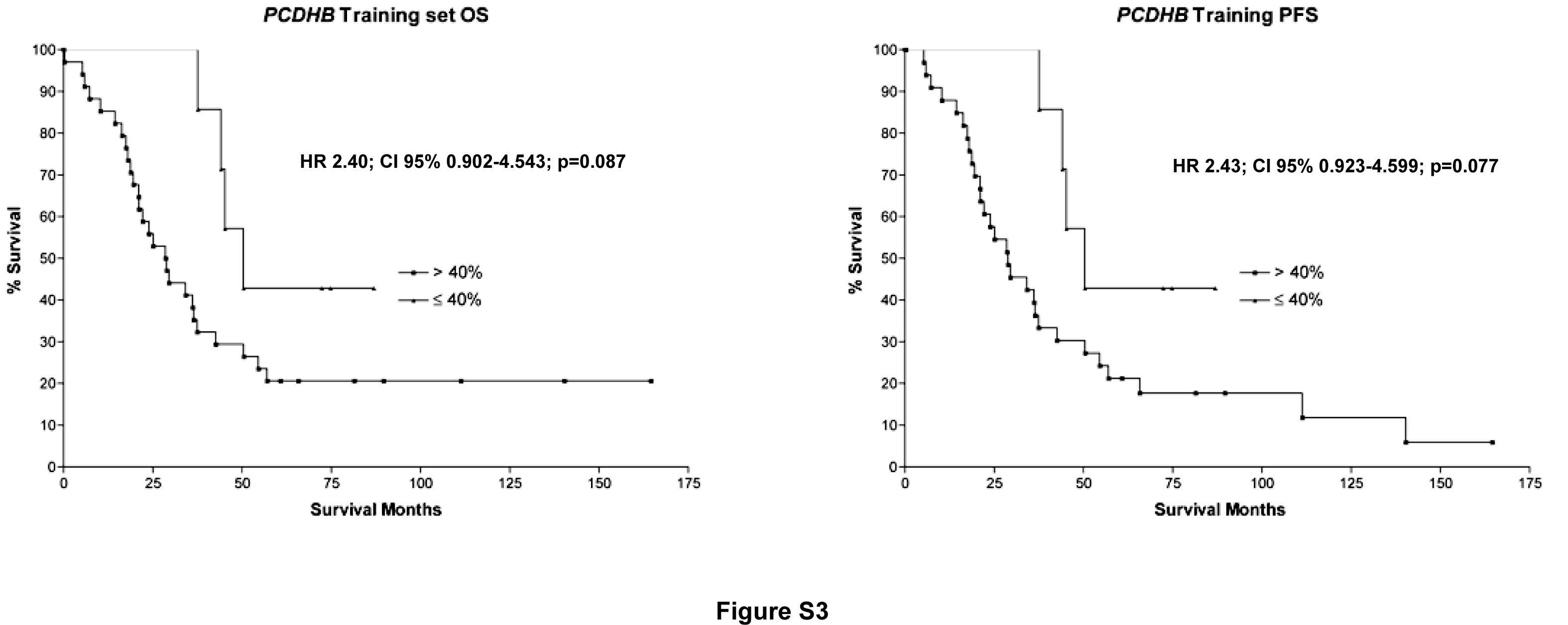

Supplement: Figure S3 — Kaplan-Meier estimates of OS and PFS of the High-Risk patients assigned to groups according to the 40% thresholds of methylation for PCDHB (Training set: ≤40% N = 7; >40 N = 34). The Hazard Ratio (HR) and the corresponding p values (Cox Long-Rank test) are reported. (TIF) [file pone.0063253.s003.tif]

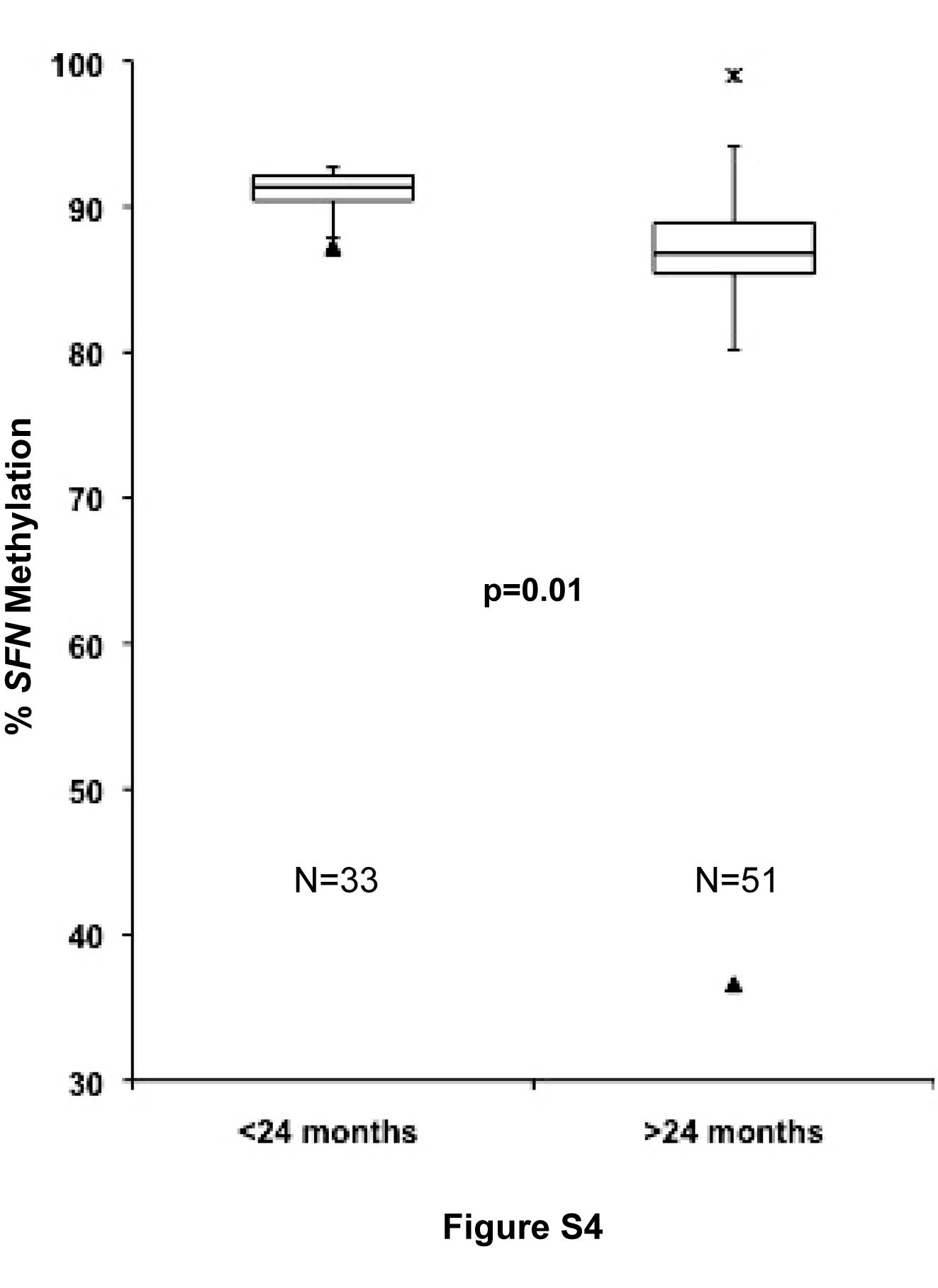

Supplement: Figure S4 — Distribution of methylation values for SFN in HR-SS patients subdivided in patients that died within 24 months and between 25 and 60 months from diagnosis. Black stars and black triangles are the upper and lower outliers, respectively. (TIF) [file pone.0063253.s004.tif]
