# Supplementary material for: Clinical Potentials of Methylator Phenotype in Stage 4 High-Risk Neuroblastoma: An Open Challenge
Source: PLoS One. 2013 May 22;8(5):e63253. doi: 10.1371/journal.pone.0063253 (PMC3661569; doi:10.1371/journal.pone.0063253)
Supplement: Table S2 — OS and PFS in High Risk stage 4 NB patients according to SFN methylation levels. (DOC) [file pone.0063253.s006.doc]

**Table S2**: OS and PFS in High Risk stage 4 NB patients according to *SFN* methylation

|  | Methylation | N | Events | Median survival (months) | HR | 95% CI | p |
| --- | --- | --- | --- | --- | --- | --- | --- |
| OS | ≤85% | 27 | 12 | 72 | 1 |  | (ref) |
|  | > 85% - ≤90% | 44 | 42 | 35.8 | 3.94 | 2.19-6.69 | < 0.0001 |
|  | > 90% | 35 | 33 | 18 | 4.73 | 2.96-10.48 | < 0.0001 |
| PFS | ≤85% | 27 | 16 | 42 | 1 |  | (ref) |
|  | > 85% - ≤90% | 44 | 43 | 24.5 | 2.86 | 1.75-5.18 | < 0.0001 |
|  | > 90% | 35 | 33 | 15 | 3.54 | 2.34-8.00 | < 0.0001 |
